# Differences in the Diets of Female and Male Red Deer: The Meaning for Sexual Segregation

**DOI:** 10.3390/biology12040540

**Published:** 2023-03-31

**Authors:** Fernanda Garcia, António Alves da Silva, Kathreen Ruckstuhl, Peter Neuhaus, Catarina Coelho, Muyang Wang, José Paulo Sousa, Joana Alves

**Affiliations:** 1 Centre for Functional Ecology (CFE), TERRA Associate Laboratory, Department of Life Sciences, University of Coimbra, 3000-456 Coimbra, Portugal; 2 Department of Biological Sciences, University of Calgary, Calgary, AB T2N 1N4, Canada; 3 State Key Laboratory of Desert and Oasis Ecology, Key Laboratory of Ecological Safety and Sustainable Development in Arid Lands, Xinjiang Institute of Ecology and Geography, Chinese Academy of Sciences, Urumqi 830011, China; 4 Sino-Tajikistan Joint Laboratory for Conservation and Utilization of Biological Resources, Urumqi 830011, China

**Keywords:** *Cervus elaphus*, sexual segregation, diet composition, forage quality, feeding behaviour

## Abstract

**Simple Summary:**

Male and female red deer typically live separately in single-sex groups throughout the year, except during the mating season when they come together in mixed groups to mate. This phenomenon is known as sexual segregation. Some experts believe that this segregation occurs because males and females have different nutritional requirements due to their distinct body sizes. Our study delves into the diets of both sexes in a Mediterranean mountainous environment and provides evidence that diet may be a contributing factor to sexual segregation. Our findings indicate that males consume more arboreal species, while both sexes primarily consume shrub species. Additionally, our research highlights the importance of evaluating other factors that may impact sexual segregation in ungulates across various species and populations.

**Abstract:**

Sexual segregation is a common phenomenon among animals, particularly dimorphic ones. Although widely addressed, the reasons and consequences of sexual segregation are still an important topic in need of better understanding. In this study, we mainly evaluate the diet composition and feeding behaviour of animals, which are related to the use of different habitats by the sexes, a special case of sexual segregation also termed habitat segregation. Sexually size dimorphic males and females often have different energetic and nutritional needs and, thus, different diets. We collected fresh faecal samples from wild Iberian red deer (*Cervus elaphus* L.) in Portugal. Samples were analysed in terms of diet composition and quality. As expected, both sexes differed in their diet composition, with males eating more arboreous species than females, but this difference was affected by sampling periods. Diet composition of both sexes had the biggest differences (and the lowest overlap) in spring, which corresponds to the end of gestation and beginning of birth. These differences might be a consequence of the sexual body size dimorphism characteristic of this species, as well as of different needs due to different reproductive costs. No differences regarding the quality of the excreted diet were observed. Our results may help to understand some patterns of sexual segregation observed in this red deer population. However, besides foraging ecology, other factors may also be contributing to sexual segregation in this Mediterranean population of red deer, and further studies focusing on sexual differences regarding feeding behaviour and digestibility are needed.

## 1. Introduction

The most well-known hypotheses of the effect of size and sex on diet composition in ruminants are based on studies of animals in captivity [1,2] and correlational studies in the field, where diets often have been assumed to be selective (availability smaller than selection) [3]. In this sense, diet differences between sexes may be due to (1) sexual body size dimorphism and sexual differences in digestive abilities [4,5] or (2) the availability and biomass of forage in their specific environments.

In sexually size-dimorphic species such as red deer (*Cervus elaphus* L.), size differences can lead to differences in nutritional needs and thus diet composition that can be the product or cause of sexual habitat segregation [6,7,8,9]. On the other hand, females and males may socially segregate within the same range or habitat [6,10]. Notwithstanding, differences in diet composition can be merely related to the different availability of food resources as a consequence of different habitat types used. Therefore, the presence of specific plants in herbivore diets may be a result of a selective process or a reflection of their abundance in the habitat in which the animals are found [11]. Selectivity is known to decrease with increasing animal densities since the availability of preferred plant species decreases [12]. Besides the chemical composition of plants, their morphology or phenology are also known as factors influencing the diet composition of herbivores [1,13].

Diet can be influenced by different physiological factors related to nutritional requirements [14,15]. Sexual body-size dimorphism causes differences in sex-specific nutritional requirements that can result in different plant selection (on a small scale) or habitat preferences (on a larger scale) [4,16]. These assumptions are based on the Jarman-Bell principle [17], which states that larger males will feed on abundant high-fibre forage, whereas the smaller females will selectively feed on high-quality, low-fibre plants [14]. Males are more efficient at digesting fibres because they have a larger rumen and a slower passage rate of food, allowing more time for digestion than the smaller females [15,18,19]. On the other hand, females may choose forages with high nitrogen, sodium, or calcium content [8] but low fibre levels, because they are less efficient at digesting fibres due to a relatively smaller stomach size and lower gut capacity compared to adult males [15,18,20]. If these assumptions are correct, females would either need to compensate for this difference in digestive ability by selecting higher quality forage than that ingested by males or by increasing their foraging efficiency [21], namely by increasing the amount of time spent foraging [22,23]. Furthermore, pregnant females likely have additional nutritional needs during gestation and lactation [15]. Although they present similar body size morphology, different nutritional requirements during these periods, besides or in addition to predator avoidance, may also lead females to segregate from non-sexually active females [4,8].

Red deer are known to consume a large variety of food, switching from shrubs and trees (being browsers) to sedges and grasses (being grazers), according to seasonal variations [1,11,24,25]. Indeed, in a review study, Gebert and Verheyden-Tixier described a total of 145 plant species that red deer may eat across Europe [24]. They are also known to consume a wide variety of fruits and seeds (e.g., acorns, chestnuts, and maize) [20,24]. They are thus classified as intermediate feeders, choosing a mixed diet while avoiding fiber (as they are not good at digesting it) [20]. To meet their nutritional needs, red deer are able to use a wide range of habitats from grasslands to forest habitats, as they provide different availability of food sources [24,26,27].

A previous study on the sexual segregation patterns of this Mediterranean population of red deer (*Cervus elaphus*) by Alves et al. (2013) [28] showed that the degree of sexual differences in habitat use was highly dependent on season. However, the study did not look at the diet composition of males and females, highlighting the need for further research. Alves et al. (2014) [29], in a previous study on this red deer population, concluded that males and females make similar use of shrubland areas. However, males and females seem to differ in their use of habitat when considering other environmental variables, such as proximity to ecotones, differences in the use of altitude, especially during the birthing period [28], or their proximity to agricultural areas or slopes [29]. They showed that the two sexes used similar land cover types, indicating that the availability of resources is similar for both males and females in terms of herbaceous and shrub species. However, for arboreal species, the larger males might be able to reach leaves higher up in the canopy that cannot be accessed by females [30].

The aim of this study was to evaluate the diet composition and quality of male and female red deer in a Mediterranean-type mountainous ecosystem. Due to previous findings, we assumed that male and female diets would differ and that these differences would be affected by age, size, and sampling periods (rut, autumn, winter, and spring). We predicted that differences in feeding composition between males and females will be more pronounced outside the rutting season when the sexes are spatially segregated, and especially during the calving season when females isolate themselves from other individuals. Due to the males’ larger body size and longer neck, we expected males to make more use of arboreous plants than females. We further predicted that females would select higher-quality plants.

## 2. Materials and Methods

### 2.1. Study Area and Study Species

The study area is the Lousã mountain located in the central region of Portugal (40°3′ N, 8°15′ W), with an approximate area of 170 km^2^ [29]. This area has a Mediterranean climate [31]. In terms of land cover, the study area is characterized by coniferous and broadleaf forests interspersed by large shrubland areas. The coniferous forests are dominated by some species of pine trees (e.g., Maritime pine (*Pinus pinaster*), Scots pine (*Pinus sylvestris*), Austrian pine (*Pinus nigra*), Douglas fir (*Pseudotsuga menziesii*), Lawson cypress (*Chamaecyparis lawsoniana*), and Mexican cypress (*Cupressus lusitanica*). In terms of broadleaf trees, the most common species are oaks (*Quercus* sp.), chestnut trees (*Castanea sativa*), Portugal laurel (*Prunus lusitanica*), common holly (*Ilex aquifolium*), ash trees (*Fraxinus* sp.), and bay laurel (*Laurus nobilis*), among others. The shrublands have a Mediterranean composition, mainly composed of heathers (*Erica* spp.), gorses (*Ulex* spp.), brooms (*Genista triacanthos* and *Cytisus striatus*), “carqueja” (*Pterospartum tridentatum*), elmleaf blackberry (*Rubus ulmifolius*), and several Gramineae species (e.g., *Agrostis castellana*, *Dactylis glomerata*, *Hordeum murinum*). Plantations of eucalyptus (mainly *Eucalyptus globulus*) are more common at the lowest elevations. The invasive species *Acacia dealbata* and *Acacia melanoxylon* are common in the study area, mostly near roads and water courses. Small agricultural fields are common near human settlements.

The red deer population in the study area dates back to a reintroduction program that took place from 1995 to 1999. The population has been growing since then, increasing in numbers and expanding in area, with an estimated density of 5.6 deer/km^2^ [26]. Although natural predators are absent, feral dogs (*Canis lupus familiaris*) prey on mainly young, subadult, and adult female deer. Furthermore, game hunting, which began in 2006, is now held on an annual basis (between October and February) outside the central part of the Lousã Mountain.

### 2.2. Data Collection

Faecal samples were collected between 2014 and 2016, in four sampling periods: rut (September to October), autumn (November to December), winter (January to February), and spring (April to May). The faecal samples were collected through direct observations of the animals defecating and directly from hunted animals in “montarias” (hunting events typical of the Iberian Peninsula), both from wild animals. Sampling methods based on direct observations and hunted animals enable the identification of the sex and age-class (calf, subadult, or adult) of each individual to which the samples belonged, without resampling the same individual. This resulted in a total of 115 unique faecal samples that were collected from males (51) and females (64).

The most abundant plant species were collected and prepared to be included in a reference collection of epidermis [32], which contained 60 plant species. The plant species were assigned to three functional groups: arboreous, shrub, and herbaceous.

### 2.3. Diet Composition Analysis

For the analyses of individual diet composition, five faecal pellets from each individual were mixed in 400 mL of water in an electric blender for 3 × 10 s pulses [25,33]. The mixture was then washed through a 0.075 mm sieve [34]. The remaining material was moved into a petri dish with sodium hypochlorite solution, which helps whiten the material [33].

We then prepared twenty microscopic slides, and identified ten plant epidermises per slide, for a total of 200 plant epidermises for each faecal sample. The quantification of the epidermis was made following systematic and alternate transects across the slide to avoid duplicating fragments [33]. The identification of epidermal fragments present in faeces was done following a reference collection of epidermis and a dichotomous key [32] with individual descriptions of each epidermal plant species.

### 2.4. Diet Quality

Nutritional quality was assessed through the quantification of the concentration of chlorophyll using absorption spectrophotometry techniques [35]. According to Christianson and Creel [35,36], the concentration of chlorophyll measured in the faeces is strongly correlated with the nitrogen content, gross energy, digestibility, and neutral detergent fibre of forage, making this a good method to estimate diet quality.

We started by drying the faecal samples at 55 °C for 24 h. Each 0.2 g subsample of faeces was boiled in 95% ethanol for 15 min. The pigment supernatant was centrifuged and then separated by decanting. This extract was evaporated (over 2 days) and reconstituted in 1 mL of 100% methanol, following a dilution of 1:31 in 100% methanol of an aliquot of 200 µL [35]. Chlorophyll concentration was likewise analysed in plants by grinding them and then applying the same protocol mentioned above. 

We performed full-spectrum scans in the Genesys 10s UV-Vis spectrophotometer on pure extracts of methanol (blanks), and on extracts from faecal and plant samples, measuring optimal density every 1 nm from 190 nm to 1100 nm, focusing on optimal density at 666 nm (peak absorption of chlorophyll) and at 750 nm (correction for turbidity) [35,36].

### 2.5. Statistical Analyses

The diet composition is expressed in terms of absolute frequency (AF) of occurrence AF = (n_i_/N_f_) × 100 and relative frequency (RF) of occurrence RF = (n_ei_/N_e_) × 100 of each plant species consumed. The n_i_ is the number of plant fragments of the species i, N_f_ is the total number of fragments in the sample (i.e., 200), n_ei_ is the number of faeces with plant fragments of the species i, and N_e_ is the total number of faeces.

A high number of different species had an AF lower than 1%, so we decided to lump these species into a group named “Other species”. The frequencies of occurrence (AF and RF) were calculated for each sex (males and females) and for the different sampling periods (rut, autumn, winter, and spring).

Differences in diet composition in terms of the three functional groups (arboreous, shrub and herbaceous), by sex and sampling period, were evaluated using ternary plots with a 2D kernel estimation and isometric log ratio transformation, suitable for compositional data. The ternary plots were generated using R Statistical Software, version 3.6.3 [37] using the “ggtern” package [38].

A two-step multivariate analysis approach was performed to evaluate differences in diet composition between males and females. In the first step, an ordination diagram (biplot) based on principal component analysis (PCA) was used to graphically visualize the differences in diet composition between the sexes. In the second step, a permutational multivariate analysis of variance (PERMANOVA) was used to test for significant statistical differences in diet composition, considering sex and sampling period as independent variables, year as a random effect, and consumed plant species as dependent variables. In PERMANOVA, all *p*-values were calculated using 9999 permutations of the residuals under a reduced model. The multivariate analysis was performed using Canoco 5, Primer 6 and Permanova+ software.

The food amplitude index (e^H′^) was also calculated, following Jost (2006) [39], where H′ is the Shannon diversity index [40]. The differences between males and females (independent variable: sex) in terms of food amplitude (e^H’^) (dependent variables) in the four sampling periods (independent variables) and their interactions were analysed using linear mixed models, where year was again included as a random effect. When significant differences were found, pairwise comparisons were performed using Bonferroni adjusted *p*-values.

To evaluate diet overlap between the sexes in each sampling period, we used the Schoener index [41]. This index varies between 0 (no food overlap) and 1 (complete food overlap).

Regarding diet quality (chlorophyll, dependent variable), a linear mixed model was used to evaluate differences between sex and sampling period (independent variables) and their interaction, with year as a random factor.

Linear mixed models were performed using IBM.SPSS, version 22. All statistical analyses were considered significant when *p* < 0.05, except in pairwise comparisons that had a Bonferroni-adjusted alpha level. The results are presented as estimated mean ± standard error (SE), unless otherwise noted.

## 3. Results

### 3.1. Diet Composition

Our results showed that shrubs were the most consumed plant group (Table 1). Considering their occurrence, it is possible to highlight the importance of *Pterospartum tridentatum*, *Ulex minor*, *Erica* spp. or *Cytisus striatus* for both male and female red deer (Table 1).

When testing for the effect of sex and sampling period on red deer diet composition, we found significant differences between the sexes (pseudo-*F*_(1, 105)_ = 3.856; *p* = 0.004) and the sampling periods (pseudo-*F*_(3, 105)_ = 9.730; *p* < 0.001), and no significant interaction between both factors (pseudo-*F*_(3, 105)_ = 1.351; *p* = 0.159). 

**Figure 1:**
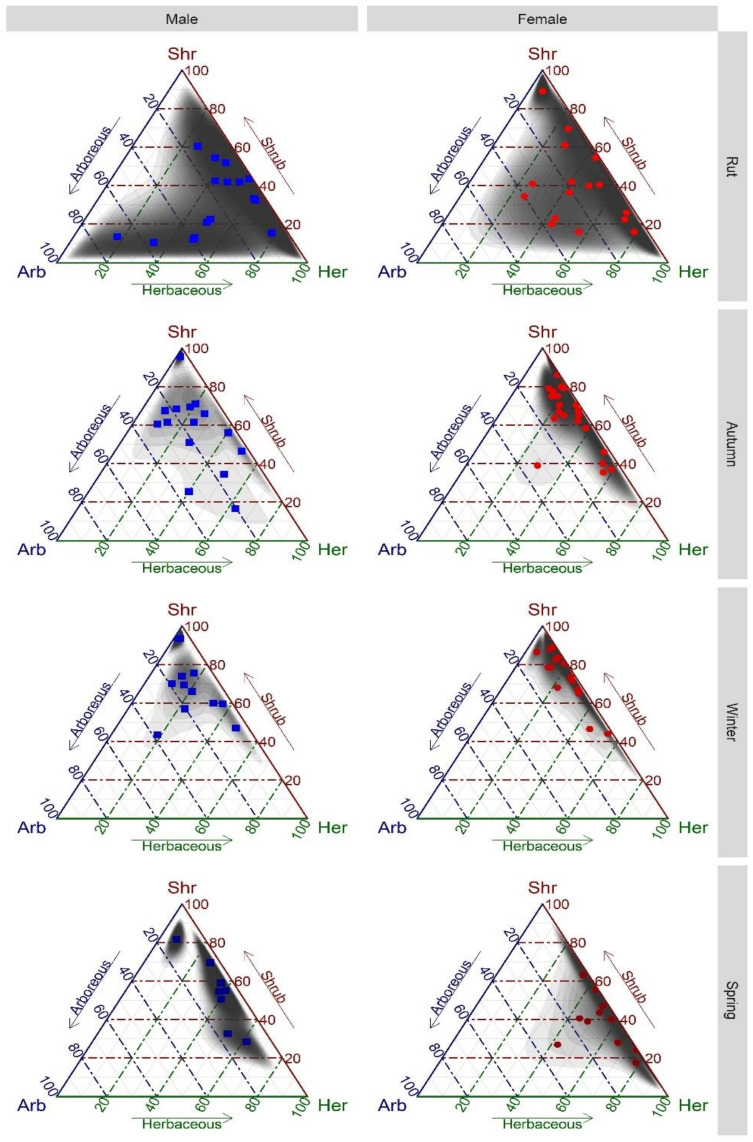
Ternary plot of the diet composition of male (blue squares) and female (red dots) red deer in each sampling period (rut (September to October), autumn (November to December), winter (January to February), and spring (April to May)). Grey shadows represent 2D kernel density estimations. Plant species were assigned to three functional groups: Arb—Arboreous, Shr—shrubs and Her—herbaceous.

Analysing the ternary plots, we found that males ate more arboreous species in all sampling periods, compared to females, except for spring, when the proportion of arboreous plants eaten by males and females was similar (Figure 1). Similarly, females had a higher proportion of shrub species in their diets, compared to males, except in spring. In spring, the biggest difference between males and females was observed in the herbaceous functional group, with females eating more herbaceous plants than males (Figure 1).

**Figure 2:**
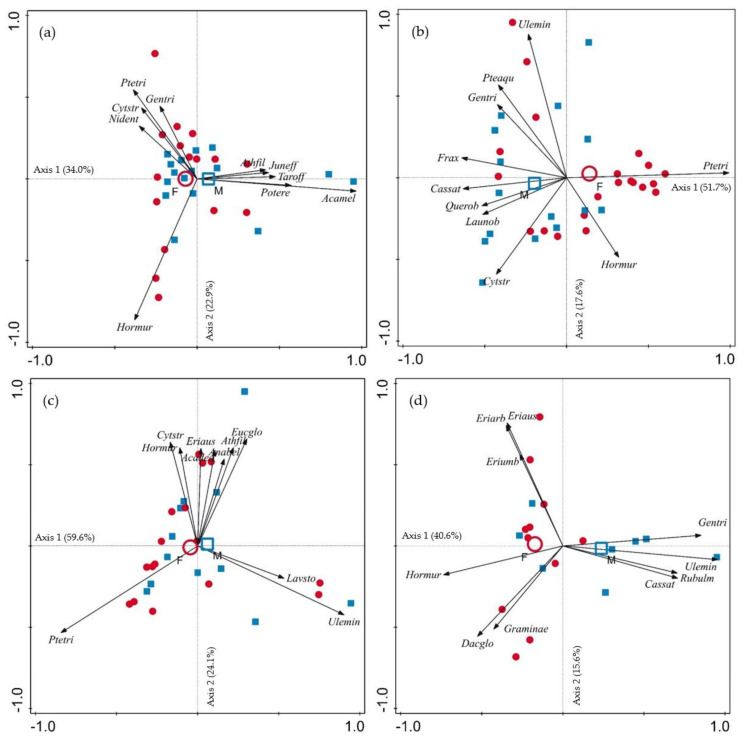
PCA biplots for (**a**) rut (September to October), (**b**) autumn (November to December), (**c**) winter (January to February) and (**d**) spring (April to May) sampling periods, showing the differences between sexes. Red circles (open and closed) represent the females (*N* = 64) and blue squares (open and closed) the males (*N* = 51). Plant species codes: Acamel: *Acacia melanoxylon*, Anabel: *Anarrhinum bellidifolium*, Athfil: *Athyrium filix−femina*, Cassat: *Castanea sativa*, Cytstr: *Cytisus striatus*, Dacglo: *Dactylis glomerata*, Eriarb: *Erica arborea*, Eriaus: *Erica australis*, Eriumb: *Erica umbellata*, Eucglo: *Eucalyptus globulus*, Frax: *Fraxinus* sp., Gentri: *Genista triacanthos*, Hormur: *Hordeum murinum*, Juneff: *Juncus effusus*, Launob: *Laurus nobilis*, Lavsto: *Lavandula stoechas*, Nidnet: Non identifiyed, Potere: *Potentilla ereta*, Pteaqu: *Pteridium aquilinum*, Ptetri: *Pterospartum tridentatum*, Querob: *Quercus robur*, Rubulm: *Rubus ulmifolius*, Taroff: *Taraxacum officinale*, Ulemin: *Ulex minor*.

Diet compositions of males and females presented higher overlap during the rut (Schoener index = 0.810; Figure 2a) and winter (Schoener index = 0.883; Figure 2c). In autumn, males consumed comparatively more *Quercus rubur*, *Castanea sativa* and *Cytisus striatus*, and females consumed more *Pterospartum tridentatum*, leading to a lower diet overlap (Schoener index = 0.687; Figure 2b). Diet overlap in spring was even lower (Schoener index = 0.661), when *Genista triacanthos* and *Ulex minor* for males, and *Erica* genus and Gramineae for females, were the most significant species in their diets (Figure 2d).

In terms of food amplitude, significant differences were found between sampling periods (*F*_(3, 101)_ = 17.710, *p* < 0.001), with the winter diet having the lowest amplitude compared to all other sampling periods (*p* < 0.001). However, there were no significant differences between the sexes (*F*_(1, 105)_ = 1.580, *p* = 0.212), or in the interaction between sex and sampling period (*F*_(3, 105)_ = 0.066, *p* = 0.978).

### 3.2. Diet Quality

**Figure 3:**
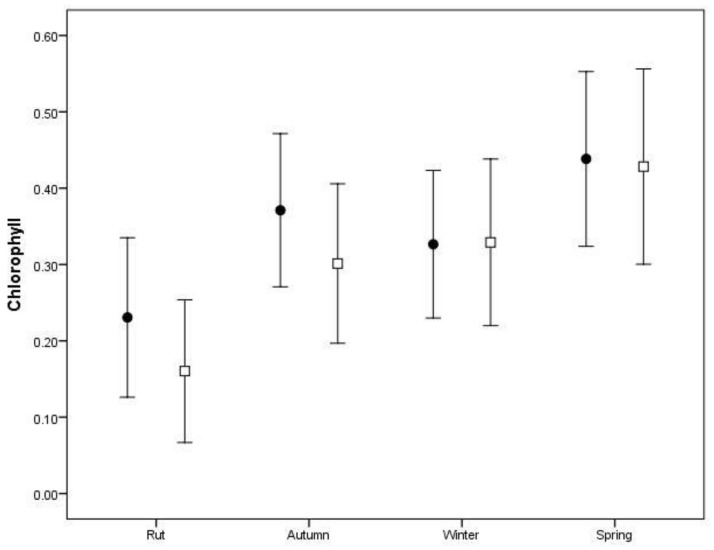
Concentration of photosynthetic pigment chlorophyll (optimal density at 666 nm) in faecal pellets of male and female red deer in each sampling period (rut (September to October), autumn (November to December), winter (January to February) and spring (April to May)). Open squares represent males (*N* = 46) and black circles females (*N* = 49).

Regarding diet quality, chlorophyll levels in the faeces were found to significantly differ between sampling periods (*F*_(3, 87)_ = 6.475, *p* = 0.001) (Figure 3). Spring faeces had the highest values of chlorophyll content (0.433 ± 0.065). However, we found no significant differences in the quality of the diet between males and females (*F*_(1, 87)_ = 0.944, *p* = 0.334), nor an interaction between sex and sampling period (*F*_(3, 87)_ = 0.269, *p* = 0.848).

In terms of plant species, arboreous species presented higher chlorophyll content (0.452 ± 0.08) than shrubs (0.261 ± 0.04) or herbaceous plants (0.253 ± 0.06).

## 4. Discussion

Our analyses showed clear seasonal differences in male and female diet composition, which were more pronounced in the autumn and spring. The proportion of arboreous species consumed was higher for males than for females, which was also found in other studies of red deer (*Cervus elaphus*) [30,42]. We found that arboreous species had higher chlorophyll levels, which corresponds to a higher quality diet. The same finding was also reported by Szemethy et al. [25] and Kamler and Homolka [43]. The consumption of a better quality diet by males was also reported for other ungulates, such as mule deer (*Odocoileus hemionus hemionus*) [44], fallow deer (*Dama dama*) [45], or mountain sheep (*Ovis canadensis nelson*) [46].

Considering the habitat used by both sexes in the study area [28,29], and the expected similar availability of forage for males and females, sexual differences in diet quality seem to be mostly explained by the increased capability of males to reach the leaves of arboreous species rather than by sexual differences in digestive ability, although we did not measure digestibility. In our population, males are 37% larger than females [26], which, in combination with their ability to stand on their back legs, makes males capable of reaching leaves that may not be reachable by the smaller females. This advantage is particularly important during the hot summers and the beginning of autumn in the Mediterranean environment. During this time, red deer can face nutritional constraints due to heat stress, with altered availability of water for herbaceous and shrub species, and thus their availability to red deer and other herbivores [2,11].

Our results support the idea that herbivore feeding ecology is influenced by seasonal changes in plant phenology [1,47], availability [11,44], and abundance [24] of preferred species. Spring and autumn were the sampling periods in which larger sexual differences in diet composition were detected. These seasonal differences may be related to the different requirements of the sexes at these specific periods of the year, or to the sexual habitat segregation exhibited during these periods [29]. Red deer males, on average, are expected to have higher energy demands than females for most of the year, since they depend on their physical condition (weight, fat reserves, and muscle) for the rut season, for competition with other males, and for antler growth [2,8,11,14]. On the other hand, spring is also an energetically challenging season for females because they are at the end of gestation and beginning of lactation after a long winter of low-quality diets [11,14]. The opposite was observed by Miranda et al. (2012) [2], where the rut was the only period in which sexual differences in diet composition were found. Although they studied the same species and the same climatic environment, their study was done on a captive herd with limited food availability and the rut occurring during the summer drought, which may explain differences in seasonal diet composition between the two studies.

Herbivores are known to adjust their feeding ecology to seasonal changes in forage availability [1,20,47], and synchronize high nutritional demands (lactation, low body fat scores, and births) to the seasons with the highest quality and availability of food [20]. While the availability of resources is greatest in the Mediterranean region in the spring, our study found that the food amplitude was not significantly higher in spring than during the rut or autumn, indicating a high selectivity by red deer (similar to what [1] found). The microhistological technique allowed us to investigate food composition, and although it is known that herbaceous plants might be underestimated, this was argued to have only a minor effect [48,49], besides being proportional to males and females. In the spring, we found a preference for *Genista triacanthos* and *Ulex minor* in males and *Erica* spp. in females, showing the strongest sexual differences in diet composition in this sampling period. However, it is also possible that males and females have different needs in terms of specific nutrients [8], and thus the differences in diet composition may reflect requirement needs rather than a strategy for an overall forage quality.

We expected that females would select higher quality forage than males because they are less efficient at digesting fibrous plants, owing to their smaller stomach size and lower gut capacity, with high rates of food passage [4,15]. On the other hand, we expected males to feed on different species, consuming more biomass instead of higher quality forage because they are capable of efficiently digesting high-fibre forages [15]. However, our results showed that males and females did not differ in terms of diet quality in either of the sampling periods, which was also found in other studies on different ruminant species [10,23]. Thus, considering the differences in diets but the lack of differences in terms of quality, males and females may have digestive plasticity and are able to retrieve their nutritional needs from different plant species [50]. Similar quality in excreted material does not entirely mean that the quality of ingested material was similar between sexes, because of significant differences regarding their digestive tracts. As we have seen, it is possible that males ingest higher quality diets since they feed more on arboreous species than females. Thus, the observed lack of differences in the quality of food found in faeces might be a result of different digestibilities in both sexes. Indeed, reproductive females are known to be able to adjust and modify the size of the small intestine to retrieve more nutrients according to their needs. This phenomenon is common between reproductive and non-reproductive females, and was verified in several species (e.g., for white tailed deer (*Odocoileus virginianus*): [50,51]). The same pattern between pregnant females and males was also observed in sika deer (*Cervus nippon*), where pregnant females had a greater gut volume and more stomach tissue than males [52]. Similar plasticity of the intestine was also found in other species of ungulates, as in the case of roe deer [53]. This kind of adaptation allows individuals to enhance nutrient uptake by increasing retention time and better processing food, especially in response to differences in diet quality [50,51,53].

Female ruminants are also known to adopt different strategies to compensate for their lower digestive efficiency, by (1) increasing mastication and feeding rates [18] or (2) increasing fermentation rate and microbial nitrogen production [4,54,55]. Indeed, in a study on captive white-tailed deer with known diets, Monteith et al. (2014) [56] showed that when males face poor diet conditions, they are unable to increase their body mass, thus being dependent on the quality of food they ingest. On the other hand, females were able to compensate when exposed to a low-quality diet by increasing absorption of dietary nitrogen and maintaining digestive efficiency [56].

According to Heape (1913) [57], males are considered “expenders of energy” and females “conservers of energy”. Females are typically caring for their dependent and vulnerable offspring, which often makes them more sedentary and reclusive, thus enhancing the probability of the survival of their offspring [8,58]. Thus, there might be a threshold between diet quality and predation risk for females [15], since they seem to be eating all available plants, regardless of the quality of each item consumed, especially if one habitat provides more predator protection than the other. In a diet study on bighorn sheep, Bleich et al. (1997) [46] argued that, given a choice, females may prefer lower predation risk over higher forage quality.

## 5. Conclusions

Our study showed that males and females differed in terms of faecal diet composition, especially in spring. Overall, compared to females, males consumed more arboreous species. The differences observed may explain some patterns of sexual segregation. However, since this was only observed with different extents in the sampling periods, regardless of the level of sexual segregation, we conclude that other factors may be involved. Considering the sexual segregation patterns of this red deer population [29] and the observed differences in diet composition in this study, we suggest that more detailed studies on sexual differences in behavioural plasticity in diets are needed from a variety of species to fully understand their habitat and nutritional requirements, particularly pertaining to sex.

## Figures and Tables

**Table 1:** Diet composition of male (*N* = 51) and female (*N* = 64) red deer in terms of absolute frequency of occurrence (AF) and relative frequency of occurrence (RF). Values indicated in bold represent the frequencies of plant groups, composed of the species that appear below them.

Plant Species/Groups	Males		Females	
	AF (%)	RF (%)	AF (%)	RF (%)
***Arboreous species***	**12.44**	**98.04**	**6.34**	**95.31**
*Acacia melanoxylon*	5.72	58.82	2.26	45.31
*Castanea sativa*	2.08	52.94	0.62	23.44
*Chamaecyparis lawsoniana*	1.30	64.71	1.33	81.25
*Fraxinus* sp.	1.47	35.29	0.77	20.31
*Laurus nobilis*	1.87	47.06	1.37	23.44
**Herbaceous species**	**30.25**	**100.00**	**32.52**	**100.00**
**-Monocots**	**27.57**	**100.00**	**30.04**	**100.00**
*Agrostis castellana*	3.48	74.51	1.93	73.44
*Dactylis glomerata*	4.71	84.31	7.41	93.75
*Hordeum murinum*	13.80	94.12	17.15	93.75
Other Gramineae	5.58	80.39	3.55	92.19
**-Other**	**2.69**	**68.63**	**2.48**	**75.00**
*Athyrium filix-femina*	1.25	66.67	1.45	60.94
*Omphalodes nitida*	1.44	47.06	1.03	67.19
**Shrub species**	**50.62**	**100.00**	**55.79**	**100.00**
*Cytisus striatus*	8.93	86.27	5.48	89.06
*Erica arborea*	1.82	72.55	2.97	81.25
*Erica australis*	2.50	76.47	2.96	85.94
*Erica umbellata*	1.44	64.71	2.73	67.19
*Genista triacanthos*	1.61	70.59	1.59	50.00
*Pterospartum tridentatum*	21.75	92.16	32.62	100.00
*Rubus ulmifolius*	1.83	45.10	0.78	45.31
*Ulex minor*	10.74	90.20	6.67	84.38
**Other Species**	**6.41**	**100.00**	**5.04**	**95.31**
**Species NI**	**0.27**	**45.10**	**0.32**	**43.75**

## Data Availability

The datasets generated and/or analysed during the current study are available from the corresponding author on reasonable request.
